# Plant Essential Oil with Biological Activity (II)

**DOI:** 10.3390/plants12203616

**Published:** 2023-10-19

**Authors:** Hazem S. Elshafie, Ippolito Camele

**Affiliations:** School of Agricultural, Forestry, Food and Environmental Sciences, University of Basilicata, Via dell’Ateneo Lucano 10, 85100 Potenza, Italy; hazem.elshafie@unibas.it

**Keywords:** aromatic plants, biochemical characterization, plant disease, pharmaceutical properties, sustainability

## Abstract

Essential oils (EOs) are concentrated hydrophobic liquids that originate from plants and contain different bioactive chemicals and volatile substances. Several plant essential oils (PEOs) are obtained from a variety of medicinal plants and have been utilized in folk medicine and traditional pharmacopoeia. They have a long history of usage as antibacterial medicines to treat various human, animal, and plant diseases. The extraction of essential oils frequently involves fractional distillation with a variety of organic solvents. EOs can be used successfully in the food and cosmetics industries in addition to their traditional use as antimicrobial agents. This Special Issue covers various significant PEOs and their individual chemical constituents and biological-pharmaceutical functions. Further information focused on the chemical characterizations, modes of action, and biopharmaceutical properties of PEOs. This Special Issue includes seventeen research papers from different geographical zones.

## 1. Introduction

Several scientific topics related to the biological activity of different plant essential oils (PEOs) have been published in this Special Issue. With regard to the seventeen papers that make up the second volume of this Special Issue, “Plant Essential Oil with Biological Activity II,” they cover several points either from chemical characterization point of view or even many biopharmaceutical properties and medical applications. The research in this volume included important essential oils (EOs) from different countries such as: *Diplosthephium juniperinum*, *Hedyosmum strigosum*, and *Dacryodes peruviana* (Ecuador); *Psidium guajava* (India); *Cupressus sempervirens* (Slovakia); *Artemisia rutifolia* EO (Russia); and *Origanum vulgare* (Italy), etc., as discussed below in detail.

## 2. An Overview of the Most Important Research

### 2.1. Southern America

In this Special Issue, three research papers about three important essential oils from Ecuador were published. In particular, a research paper was carried out by Salinas et al. [1] to biochemically characterize the EO extracted from *Diplosthephium juniperinum* in Ecuador. The results of this research showed moderate inhibitory effects regarding the acetylcholinesterase and butyrylcholinesterase enzymes and also low antioxidant activities, whereas another paper was carried out by Cartuche et al. [2] for studying the biological activity profiling of *Hedyosmum strigosum* EO, an aromatic native shrub from southern Ecuador. The results of this research demonstrated that the main compounds of this EO were thymol, -phellandrene, thymol acetate, and linalool, accounting for more than 51% of the EO composition. In addition, *H. strigosum* EO showed strong antioxidant and antimicrobial activities and moderate acetylcholinesterase inhibitory effects. Another study was carried out by Espinoza et al. [3], who investigated the in vivo anti-inflammatory efficacy of the copal (*Dacryodes peruviana*) EO native species from Ecuador. The results showed a moisturizing effect and an alleviation of several events occurring during the inflammatory process after topical treatment with the EO, such as a decline in skin edema, a reduction in leukocytic infiltrate, and a decrease in inflammatory cytokines, and hence they concluded that this EO could be an attractive treatment for skin inflammation.

### 2.2. Middle East

Several studies have been also carried out on some important EOs from the Middle East region, especially Egypt and Saudia Arabia, as follows: (i) Eos of *Jatropha intigrimma*, *J. roseae*, and *J. gossypifolia* (Egypt), which was carried out by Gamal El-Din et al. [4]; (ii) EO of *Devrra triradiata* (Saudi Arabia), carried out by Guetat et al. [5]; (iii) EO of *Acacia nilotica* (Egypt), carried out by El Gendy et al. [6]; (iv) EO of *Thyme vulgaris* (Egypt) carried out by Abd-Ellatif et al. [7]; (v) EOs of six different cultivars of *Citrus reticulata* (Egypt), carried out by Fahmy et al. [8]; and (vi) volatile EOs extracted from aerial parts of male and female ecospecies of *Ochradenus arabicus* (Saudi Arabia), carried out by Abd-ElGawad et al. [9].

In addition, this Special Issue also contained a study carried out by Khan et al. [10] on the potential antimicrobial and anticancer properties of seed extracts from *Citrullus colocynthis* (Saudia Arabia) by using different organic solvents, such as methanol, hexane, and chloroform. 

This Special Issue also included important research on the comparative metabolic study of *Tamarindus indica* (Egypt) from various organs (bark, leaves, seeds, and fruits) and evaluated their anti-inflammatory effects. This study was carried out by Aly et al. [11] and concluded that the tested extracts from various organs of *T. indica* showed considerable anti-inflammatory and wound-healing activities.

### 2.3. Western Africa

One research paper has been published also in this volume regarding an EO from Northwest Africa. The study was carried out by Benali et al. [12], who studied the chemical profiling and biological properties of EOs extracted from *Lavandula stoechas* collected from three Moroccan sites. The chemical GC/MS profiles of the three studied EOs indicated that their biosyntheses varied depending on the site of growth. The studied EOs have also explicated promising antibacterial activities against Gram-positive and Gram-negative bacteria such as *Bacillus subtilis* and *Pseudomonas aeruginosa*. These important biological characteristics of *L. stoechas* EOs proved that this plant is a valuable source of naturally occurring bioactive chemicals with therapeutic effects.

### 2.4. Eastern Asia

Another study in this Special Issue focused on the chemical composition, antimicrobial effect, and anticancer activity of an EO extracted from *Psidium guajava* (India). This research was carried out by Alam et al. [13]. The GC–MS revealed that this EO was composed mainly from limonene and caryophyllene and concluded that this EO has promising antimicrobial and anticancer activities and could be a useful source for developing a natural therapeutic agent for oral infections and oral cancer.

### 2.5. Eastern Europe

The second volume of this Special Issue also included some important research papers from Europe. Among them, Galovičová et al. [14] evaluated the antioxidant, antibiofilm, antimicrobial (*in situ* and *in vitro*), insecticidal, and antiproliferative activities of *Cupressus sempervirens* EO (Slovakia). They concluded that *C. sempervirens* could be a suitable natural alternative as a biocontrol agent against different types of microorganisms, as well as suitable for controlling biofilms and harmful agricultural pests.

Dylenova et al. [15] studied the chemical diversity of *Artemisia rutifolia* EO (Russia) and evaluated its antimicrobial and antiradical activities. Their results showed that this EO can be classified into Tajik and Buryat-Mongol chemotypes and has strong antimicrobial activity against Gram-positive bacteria and fungi and high antiradical activity. The authors of this study concluded that the EO from *A. rutifolia* in the Russian flora indicates the prospects of the species as a raw material for the pharmaceutical and cosmetic industry. 

### 2.6. Western Europe

Zinno et al. [16] studied the chemical composition and biological activities of two *Origanum vulgare* genotypes widely cultivated in Sicily (Italy). Their results demonstrated that these studied EOs have high antimicrobial activities, both in vitro and in a food matrix challenge test. These results suggested their potential use as biocontrol agents against a wide spectrum of foodborne pathogens.

## 3. Conclusions

As a result, the studies in this Special Issue demonstrated that several examined PEOs had positive potential for use in many bio-pharmacological applications, as well as in the agriculture and food sectors. Many studies in this issue emphasized the potential uses of a variety of PEOs in the agri-food industry, where they exhibit promising antimicrobial activities against a wide range of food deterioration microorganisms and prolong shelf-lives of processed food. Additionally, the investigated EOs and their primary components have been employed successfully as potential natural substitutes for synthetic drugs against a number of phytopathogens. Numerous researchers have studied the mechanisms of biological ability and have linked this potentiality to a distinctive chemical makeup, which is mainly composed of terpenoids and phenolic chemicals.
